# URI-CADS: A Fully Automated Computer-Aided Diagnosis System for Ultrasound Renal Imaging

**DOI:** 10.1007/s10278-024-01055-4

**Published:** 2024-02-27

**Authors:** Miguel Molina-Moreno, Iván González-Díaz, Maite Rivera Gorrín, Víctor Burguera Vion, Fernando Díaz-de-María

**Affiliations:** 1 Department of Signal Theory and Communications, Universidad Carlos III de Madrid, Avda. de la Universidad, 30, Leganés, 28911 Spain; 2 Hospital Ramón y Cajal, M-607, 9, 100, Madrid, 28034 Spain; 3 Instituto Ramón y Cajal de Investigación Sanitaria (IRyCis), Ctra. Colmenar Viejo, Madrid, 28034 Spain; 4 Universidad de Alcalá, Pl. de San Diego, s/n, Alcalá de Henares, 28801 Spain

**Keywords:** Ultrasound renal imaging, Computer-aided diagnosis, Pathology detection, Machine learning, Convolutional neural networks

## Abstract

Ultrasound is a widespread imaging modality, with special application in medical fields such as nephrology. However, automated approaches for ultrasound renal interpretation still pose some challenges: (1) the need for manual supervision by experts at various stages of the system, which prevents its adoption in primary healthcare, and (2) their limited considered taxonomy (e.g., reduced number of pathologies), which makes them unsuitable for training practitioners and providing support to experts. This paper proposes a fully automated computer-aided diagnosis system for ultrasound renal imaging addressing both of these challenges. Our system is based in a multi-task architecture, which is implemented by a three-branched convolutional neural network and is capable of segmenting the kidney and detecting global and local pathologies with no need of human interaction during diagnosis. The integration of different image perspectives at distinct granularities enhanced the proposed diagnosis. We employ a large (1985 images) and demanding ultrasound renal imaging database, publicly released with the system and annotated on the basis of an exhaustive taxonomy of two global and nine local pathologies (including cysts, lithiasis, hydronephrosis, angiomyolipoma), establishing a benchmark for ultrasound renal interpretation. Experiments show that our proposed method outperforms several state-of-the-art methods in both segmentation and diagnosis tasks and leverages the combination of global and local image information to improve the diagnosis. Our results, with a 87.41% of AUC in healthy-pathological diagnosis and 81.90% in multi-pathological diagnosis, support the use of our system as a helpful tool in the healthcare system.

## Introduction

Ultrasound (US) is one of the most versatile and widely use medical imaging techniques due to its advantages such as low-cost, real-time operation ability, and lack of ionizing radiation [1]. Although US imaging is used in many medical fields (abdominal, fetal, etc.), it still poses major challenges to interpretation due to several factors, such as varying pressure applied to the emitter and level of gain, speckle noise, shadows caused by hyper-echoic areas, or boundary ambiguities [2].

In particular, US imaging is the prevalent technique for visualizing kidneys in nephrology. Nevertheless, in clinical practice, it shows a significant inter- and intra-practitioner variability, and its interpretation for diagnosis purposes is challenging. This has prompted the creation of reference texts, such as the comprehensive atlas written by O’Neill [3], which provides descriptions of various kidney abnormalities in US images. As a result, the current approach requires training experts to interpret US renal images, which limits the task scope to specialists.

On the other hand, computer-aided diagnosis (CAD) systems have emerged as one of the areas of significant interest for the medical community [4]. In the field of nephrology, standard CAD systems still rely on traditional image descriptors: Haar [5] or Gray Level Co-ocurrence Matrix, GLCM [6], which are fed to a classification algorithm, for both segmentation and diagnosis of the kidney [7, 8]. Convolutional neural networks (CNNs) have shown significant potential in other US imaging tasks such as breast nodule classification [9, 10], thyroid nodule detection and classification [11, 12], or diagnosis of focal liver lesions [13]. However, in the case of US renal imaging, the lack of large and annotated datasets hinder the training of CNN-based CAD systems. The existing CAD systems in this field either rely on small datasets [14, 15] or do not tackle the full problem of segmentation and diagnosis of a complete taxonomy of pathologies [16–19].

In this paper, we present URI-CADS, a fully automated computer-aided diagnosis system for ultrasound renal imaging. To the best of our knowledge, this is the first attempt to simultaneously segment and perform a comprehensive characterization of a complete kidney pathology taxonomy in a real scenario and to establish a benchmark in this field. The main goal of the system is twofold: (1) it can be used in primary healthcare by non-expert practitioners to filter out clinical cases that need to be referred to specialists, improving clinical workflow and reducing specialist workload; and (2) due to the comprehensive set of pathologies it addresses, it can serve as a useful tool for training practitioners and supporting experts. For that purpose, we have developed a robust framework, based on Mask-RCNN [20] and Faster R-CNN [21], able to detect areas of interest and fuse global and local information to perform a tentative diagnosis of the images, which enables the medical community to gain some insights into the different pathologies of the clinical cases.

As we will show in the experimental section, our approach segments the kidney and provides a complete tentative diagnosis that can offer valuable insights to practitioners in their daily activity. Moreover, the fact that the system considers both global and local pathologies in the diagnostic process improves performance and identifies areas of interest that should be analyzed by experts for the final diagnose of the case.

We next detail the main contributions of our work:

- We introduce a fully automated computer-aided diagnosis system for US renal imaging, which seamlessly integrates segmentation, detection of areas of interest, and global diagnosis into a single architecture.
- The model incorporates a segmentation task as a regularizer for the main classification tasks, resulting in an improved performance during diagnosis.
- The system integrates image- and region-based analyses at multiple resolutions to enhance the performance by jointly leveraging the advantages of both perspectives at different granularities. This multi-perspective approach has been shown to yield much better results than those of several state-of-the-art methods.
- The proposed system has the ability to provide two complementary diagnoses: a binary (healthy vs. pathological) diagnosis and a multi-class diagnosis with two global categories (hyper-echoic cortex and poor corticomedullary differentiation) and four local categories (cyst, stone, hydronephrosis, and others). The local ones can be easily expanded to nine classes if the database is extended accordingly.
- We additionally release our database, which will become the first public benchmark in the field of diagnosis from US renal imaging, promoting the advancement of knowledge in the field and contributing to the improvement of diagnosis and existing therapies.

The remainder of this paper is organized as follows: “Related Work” section briefly reviews the related literature. In “Method” section, we first provide the details about our data acquisition process and then a general description of our method for segmentation and diagnosis of renal US imaging, followed by a more detailed description of each module. “Results” and “Discussion” sections describe and discuss the experimental results that support our method, respectively, and, finally, in “Conclusion” section, we summarize our conclusions and outline future lines of research.

## Related Work

In this section, we briefly describe the state-of-the-art methods for kidney US segmentation and classification found in the literature, and we compare our approach with the most recent methods for biomedical disease detection.

### 2D Kidney Ultrasound Segmentation

The lack of open, annotated, and large-scale datasets in 2D kidney US segmentation hinders the performance comparison among the methods in the literature. Consequently, all the literature results presented in this section are accompanied by the number of US images in the test set.

Traditional approaches for kidney automatic segmentation have handled the ill-posed nature of the segmentation problem using supervised algorithms based on conventional features or level-set methods. Vaish et al. proposed to crop the rectangular region that contains the kidney by adapting the cascade classifier by Viola-Jones [5], which uses the AdaBoost algorithm over Haar features to detect faces [7]. The main disadvantage of this approach is that rectangular regions are not expressive enough to represent properly the major and minor axis of the kidney, which are variables correlated with some illnesses. Other methods relied on energy minimization of some image properties (gradients, curvatures, etc.) to generate a pixel-wise segmentation of the kidney [22, 23]. Zheng et al. proposed a new graph-cut-based method to segment kidney US images by integrating image intensity information and texture feature maps extracted using Gabor filters [24]. However, all these approaches are computationally expensive, and they have been assessed using test sets restricted to tens of US images, which strongly limits the significance of the results.

Regarding the CNN-based methods, some were focused on learning the shape and boundaries of the kidney. Ravishankar et al. proposed a generative model of image formation to jointly learn the appearance, i.e., texture (foreground and background) and the kidney shape for US kidney segmentation [25]. They proposed the use of U-Net [26] with a loss function that models the contextual interactions of foreground and background with shared parameters. The proposed architecture obtained a 8% improvement (reaching 74%) in terms of the Dice coefficient with respect to the baseline system, in a test set composed of 131 US images. Additionally, the same authors used a shape-regularization (SR) network to complete the failure modes of a FCN, i.e., the low-quality segmentations [27]. The best results (84%) were obtained with a complex setup when the SR network was pre-trained with predictions sampled in different epochs before convergence, and the weights of the first network were updated with the results of back-propagating a custom loss which made use of both the preliminary and shape-regularized predictions and the encoded predictions obtained from the low-dimension bottom layer of U-Net. In this case, the test set consisted of 171 US images. A recent approach by Chen et al. proposed a multi-scale fusion network of structural features (with a boundary detection module) and detailed features (SDFNet) to extract structural features, capture texture details, and merge features, respectively [28]. The mean Jaccard coefficient in a test set of 50 US images was 91%.

Another set of approaches used pretraining in natural image databases, or ad-hoc databases created for this purpose. In particular, Deepthy et al. used a backbone pretrained in ImageNet [29] as a basis for training the system on 560 US images in [30]. The resulting Dice coefficient was 62%. Yin et al. first used deep neural networks pretrained for classification in ImageNet to extract high-level image features from US images. These features were used as input to learn kidney boundary distance maps using a boundary distance regression network, and the predicted boundary distance maps were classified as kidney pixels or non-kidney pixels using a pixel-wise classification network in an end-to-end learning fashion. In 289 US images, the Jaccard coefficient reached a value of 87% [31]. Finally, Song et al. adopted a cycle generative adversarial network (CycleGAN) to synthesize US images from CT data and construct a transition dataset to mitigate the immense domain discrepancy between US and CT. Mainstream convolutional neural networks were pretrained on the transition dataset and then transferred to real US images. They tested their approach over two sets of 30 and 82 US images, achieving a Dice coefficient of 95% and 87%, respectively [32].

### 2D Kidney Ultrasound Classification

Kidney US classification has been traditionally tackled in two steps, feature extraction and classification. In particular, some features, such as statistics over the Gray Level Co-ocurrence Matrix (GLCM) or the histogram, have been broadly used for pathology detection in renal ultrasound. Due to the fact that many of the kidney pathologies (as cysts of stones) appear in the images as hypo- or hyper-echoic areas, these features are useful to describe the texture and gray-level distribution in the images. Krishna et al. used these kinds of features as an input for a SVM classifier to distinguish between healthy-stone/cyst kidney images [33]. Other approaches used a k-NN classifier to classify healthy and cystic images [34]. Attia et al. increased the taxonomy to healthy, cyst, stone, tumor, and renal failure and used a neural network classifier [35]. However, all of those methods were tested in only tens of images, and given that statistics over the GLCM matrix or the histogram are prone to overfit small sets of data, their generalization to more demanding and complete datasets is not proven.

In recent years, the research trend based on CNNs has also focused on US kidney classification. Texture- and gray level-based features do not take into account the global shape of the lesions, their position inside the kidney, or complex relationships between parts of the image. In this sense, the task can leverage the CNN ability to extract complex relationships among different areas of the image.

Regarding CNN-based methods, Shi proposed a hybrid deep learning architecture for accurate kidney injury prediction, with patient data and US kidney images as input. Its reported accuracy was 90% on a test set of 122 images, but the system uses an ensemble of three different CNNs [18]. Another recent approach by Smail et al. used a five layer CNN over 2.420 sagittal hydronephrosis US images to grade their severity. They obtained a classification accuracy of 51% [19]. Finally, Sudharson and Kokil proposed an approach similar to the one presented in this paper, where the predictions of three different CNNs were combined as input to an SVM to distinguish four categories of kidney images: normal, cyst, stone, and tumor [16, 17]. Their best reported accuracy is 95%, but over a set of high-quality images (selected from an original database) corrupted by synthetic speckle noise to generate a test set of 520 augmented images.

Inspired by this kind of approaches, in this paper, we propose to develop a framework to jointly segment and provide a preliminary diagnose to 2D kidney US images. Although other approaches have tried to diagnose different pathologies in US renal images, to our knowledge, this is the first attempt to simultaneously segment and perform a comprehensive characterization of a complete kidney pathology taxonomy in a real US scenario. Other very recent approaches have performed this kind of study in tomography images, with radiation exposure and higher cost, but significantly better resolution (it is, therefore, a less demanding scenario). For example, Özdaç et al. classify 3 different retinal diseases in optical coherence tomography (OCT) images [36], and Uysal detects monkeypox in skin images [37]. In the field of kidney disease detection, in [38], they diagnose chronic kidney disease (CKD) with histopathological images with an AUC of 96.3% in 2935 patients, but the procedure is invasive, as it requires a biopsy. Lastly, the methods described in [39, 40] classify large sets of 2D computed tomography (CT) kidney images (1812 and 12,664, respectively) into healthy, cyst, stone, and tumor. Their reported accuracy is 99.8% and 82.52%, respectively.

In contrast to these approaches, we have developed a robust framework for low resolution US images, based on Mask-RCNN [20] and Faster R-CNN [21], able to detect areas of interest and fuse global and local information to perform a tentative diagnosis of the images. Our system enables the medical community to gain some insights into the different pathologies of the clinical cases with a low-cost, non-invasive, and risk-free imaging technique. Furthermore, the nonexistence of a benchmark for US renal imaging hinders the performance comparison among the state-of-the-art approaches, unlike other imaging modalities, such as 2D CT renal imaging [41, 42]. We expect our dataset to become a benchmark in the field of US renal imaging.

## Method

In this section, we first describe the problem and the taxonomy of pathologies addressed by URI-CADS, our computer-aided diagnosis system for ultrasound renal imaging. Then, we provide a general description of our fully automated system and subsequently a detailed explanation of its constituent processing blocks in the following subsections.

### Patient Population and US Image Acquisition

A total number of 1985 sex-balanced US B-mode renal images were collected retrospectively, with 450 healthy and 1535 pathological kidneys, from patients over 18 years of age. Left and right kidneys are also balanced in the collection, and both transversal (93%) and longitudinal (7%) images are present. Images were anonymized to ensure that they do not contain any personal information that could lead to the identification of the patients and were collected during the years 2009 and 2018 at the rate of one image per clinical case.

Images were acquired through a Toshiba Xario-660a ecographer with 3MHz and 3.5MHz convex multi-frequency probes and different capture parameters: field of view, zoom, etc. The varied collection of images were written in JPG format, with variable sizes ranging from $[375-600,382-810]$ height-width pixels.

### US Image Annotation and Interpretation

A US renal image depicts a kidney (either in transversal or longitudinal position) which may exhibit pathologies at two different levels: global and/or local. This hierarchical point of view, with two different levels of granularity, is inherent to the interpretation of the US kidney image and provides valuable information.

**Fig. 1:**
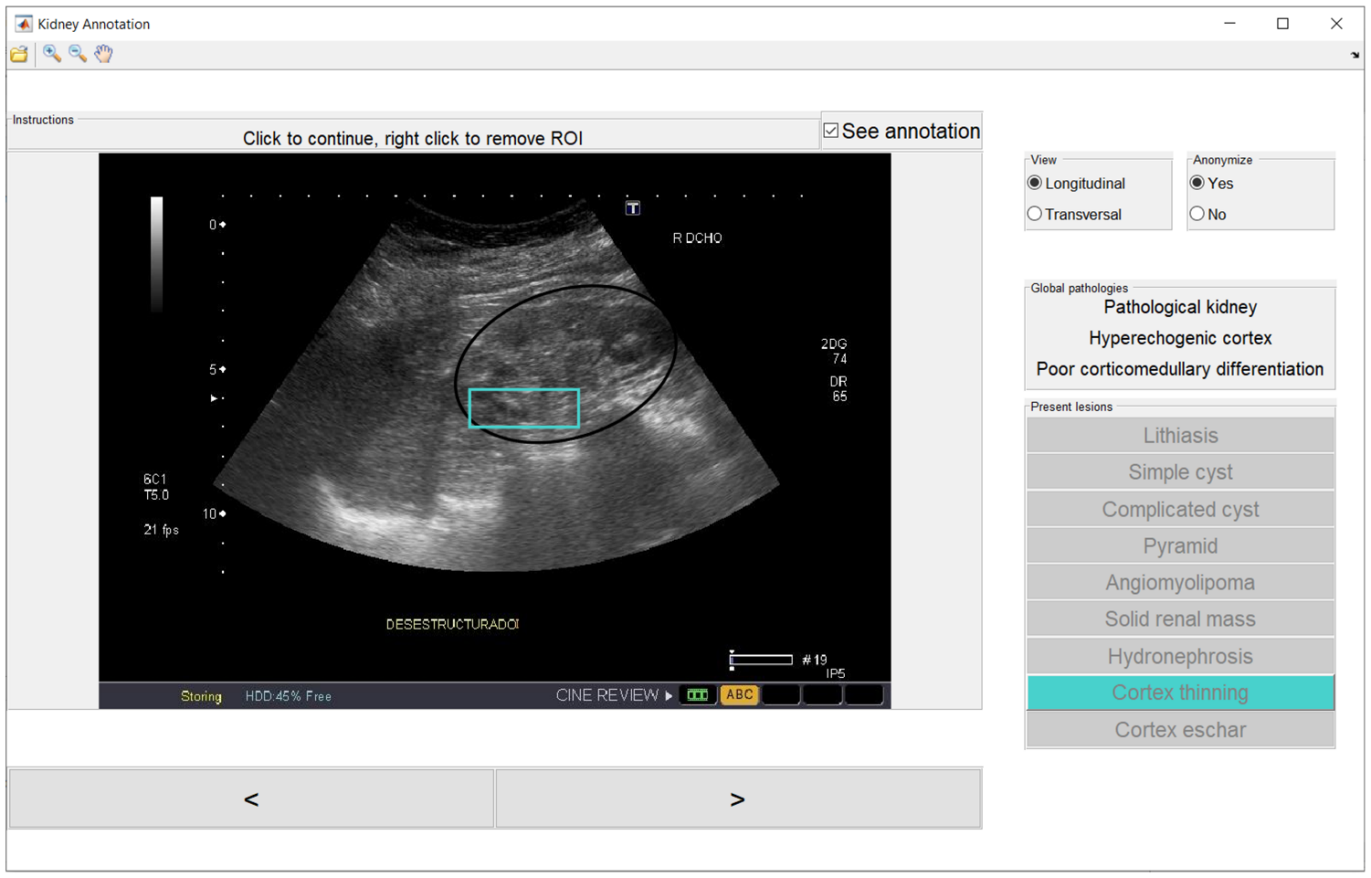
Illustrative example of the annotation of an image of the database

Two experienced nephrologists (M. R. G. and V. B. V., with 25 and 10 years of experience in US renal interpretation, respectively) from Hospital Ramón y Cajal, Madrid (Spain), have independently annotated each clinical case, and consensus was reached by discussion in the event of disagreement. Both facultatives were blinded to the patient record. The annotation process has been performed manually through an ad-hoc annotation application. The annotation of each image includes the following fields: an associated segmentation mask of the kidney (a polygonal segmentation delineated over an ellipse drawn by the nephrologists); an indicator of whether it contains global pathologies and, if so, which ones; and, if present, the bounding box coordinates of the local lesions and their indicators. An example of the annotation is shown in Fig. 1.

**Fig. 2:**
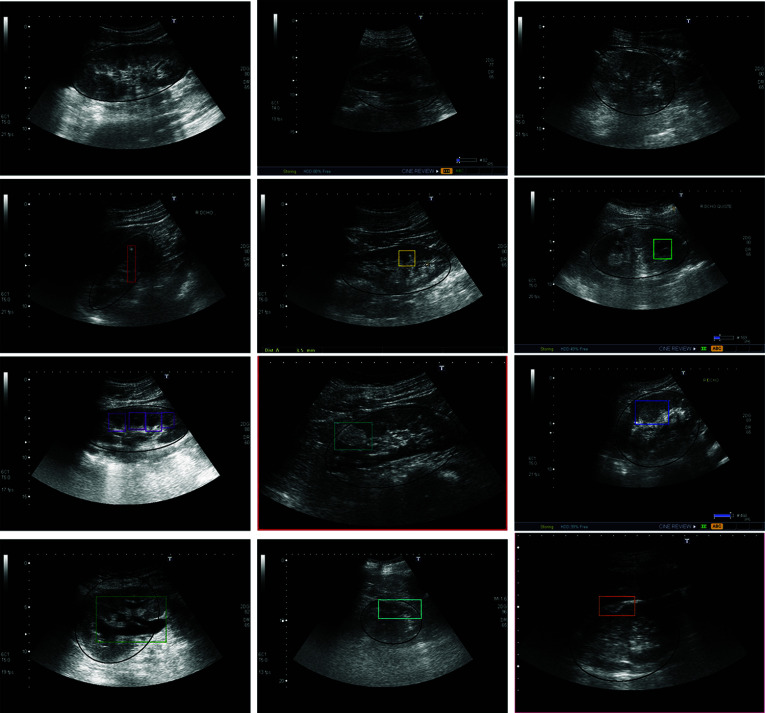
Illustrative examples of several global and local pathologies. Black: kidney location. Colored bounding boxes: local pathology locations. From left to right, first row: healthy kidney, poor corticomedullary differentiation, and hyper-echoic renal cortex; second row, lithiasis, simple cyst, complicated cyst; third row: pyramids, angiomyolipoma, solid renal mass; and fourth row: hydronephrosis, cortex thinning, and cortex eschar

Table 1 summarizes the complete taxonomy of pathologies considered in this paper, designed by the two expert nephrologists. In summary, they propose a set of 2 global categories: poor corticomedullary distinction and hyper-echoic renal cortex; 9 local pathologies: simple and complicated cysts, hydronephrosis, pyramids, lithiasis, angiomyolipoma, solid renal mass, cortex thinning, and cortex eschar; and an additional category for healthy kidneys. In addition, Fig. 2 shows a representative example for each one of the considered pathologies.

**Table 1:** Taxonomy of the considered global and local pathologies with their description. For practical purposes, we distinguish among seven categories: healthy (H), poor corticomedullary distinction (PCD), hyper-echoic cortex (HC), cyst (C), pyramid (P), hydronephrosis (HYD), and others (O)

**Category acronym**	**Taxonomy acronym**	**Type**	**Description**
**H**	**H**	Global	**Healthy kidney**. Two concentric parts are distinguished: renal cortex, the darker external part, and renal sinus, the brightest internal part.
**PCD**	**PCD**	Global	**Poor corticomedullary distinction.** In this case, renal cortex and sinus can not be distinguished correctly.
**HC**	**HC**	Global	**Hyper-echoic cortex.** Renal cortex is hyper-echoic, which causes a low contrast in the internal part of the kidney.
**C**	**SCY**	Local	**Simple cyst**. Simple cysts are usually hypo-echoic (darker), uniform, and spherical areas within the kidney.
	**CCY**	Local	**Complicated cyst**. Complicated cysts are very similar to simple ones, but can have a less uniform texture.
**PYR**	**PYR**	Local	**Pyramid**. Pyramids are kidney areas with a regular position, between renal cortex and sinus, and, if they are hypo-echoics may be a symptom of chronic kidney disease. They usually have a less spherical shape than the cysts.
**HYD**	**HYD**	Local	**Hydronephrosis**. Hydronephrosis is a difficulty to remove the urine. Hence, the urine provoques hypo-echogenia in renal sinus, and in many cases, that the urine via becomes visible.
**O**	**LIT**	Local	**Lithiasis**. Lithiasis appears as a hyper-echoic area (brightest) in the internal part of the kidney that shades a part of the image in the direction of ultrasound capture.
	**ANG**	Local	**Angiomyolipoma**. Angiomyolipoma is a benign tumor that appears as a hyper-echoic area in the US image, generally in renal cortex.
	**SRM**	Local	**Solid renal mass**. It is a possibly malignant tumor that is hypo-echoic in appearance and is not easy to distinguish from cysts.
	**CT**	Local	**Cortex thinning.** Renal cortex reduces its thickness in a specific part of the contour of the kidney.
	**CE**	Local	**Cortex eschar**. Renal cortex has scars in some areas; it is not uniform.

### General Overview of URI-CADS

**Fig. 3:**
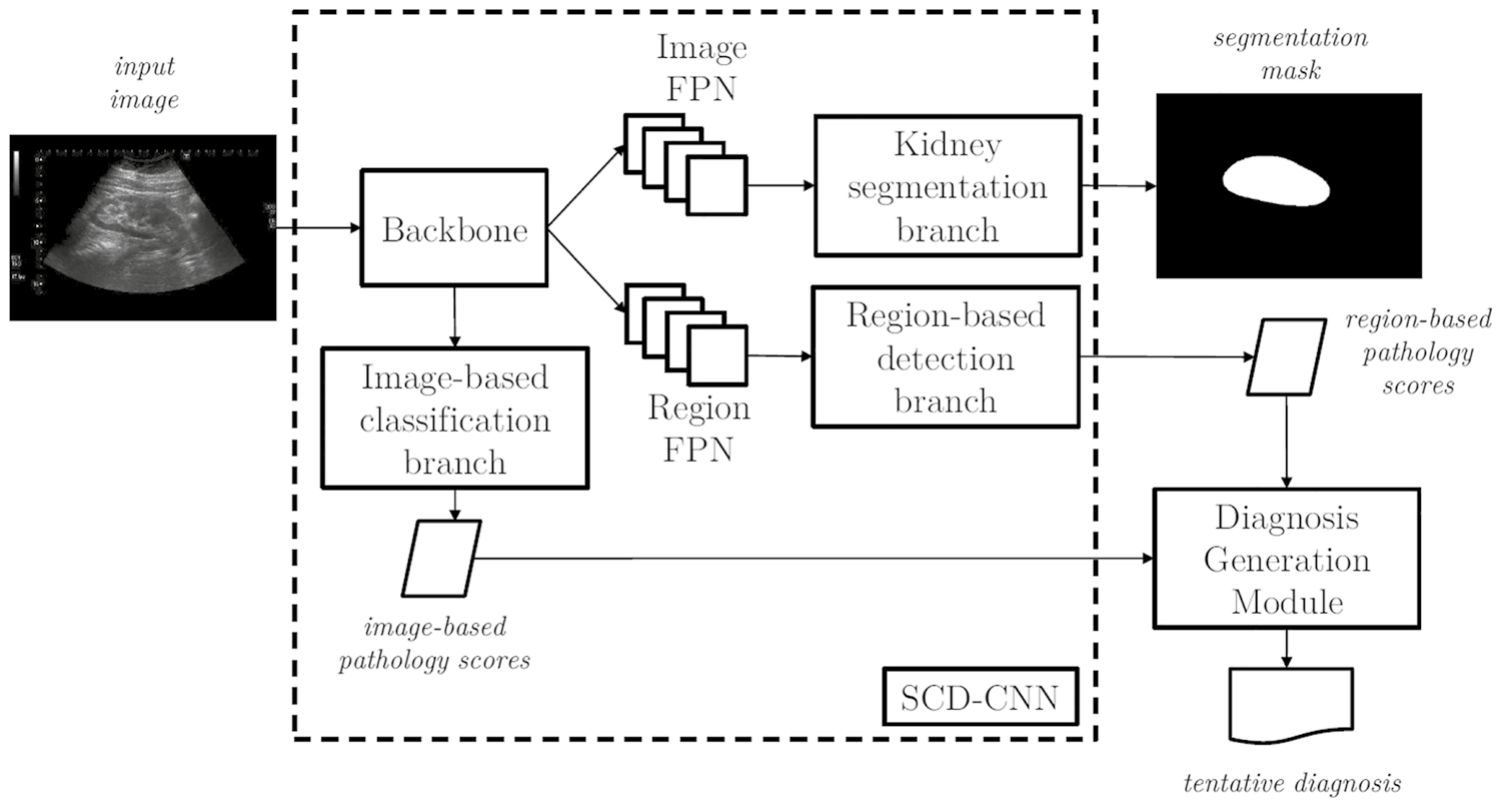
Processing pipeline of URI-CADS. Each 2D US image is fed into the automated system, which produces a kidney segmentation mask, an image-based set of global and local predicted pathologies and region-based proposed local pathologies with their locations. Then, the region-based pathology scores are combined with those coming from the image-based classification branch to generate the tentative diagnosis

The high-level pipeline of URI-CADS is depicted in Fig. 3. The architecture can be divided into two main blocks: the first one is called *SCD-CNN* (where SCD stands for segmentation, classification, and detection) and is responsible for obtaining the kidney segmentation mask and pathology predictions from both image- and region-based approaches, and the second one is the *Diagnosis Generation Module*, which, from the description provided by the SCD-CNN, combines the predictions coming from image- and region-based perspectives to provide a tentative diagnosis for each clinical case.

**Fig. 4:**
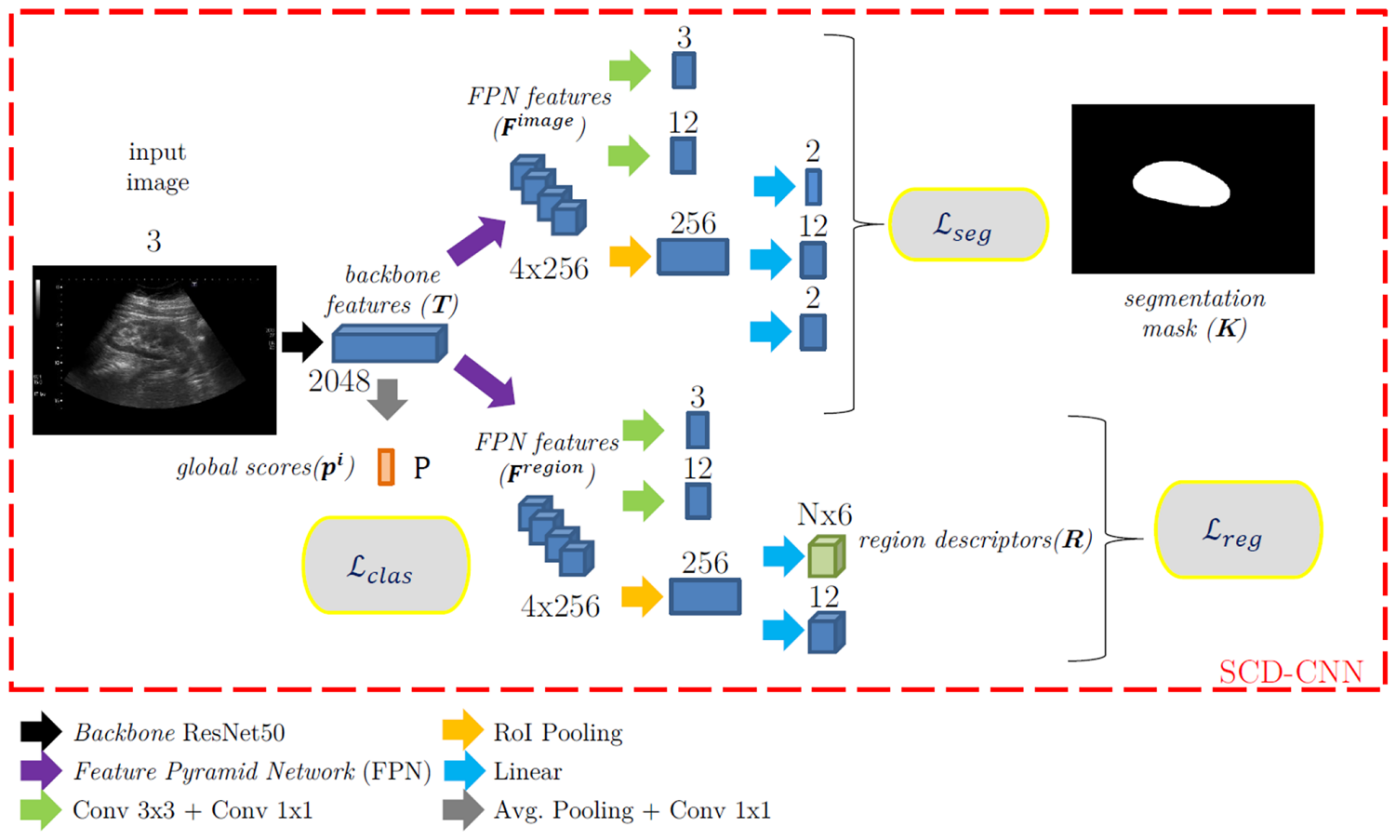
Architecture of the SCD-CNN and the loss functions employed for training. It is composed of three branches: one for kidney segmentation, one for image-based pathology detection, and one for region-based pathology detection; and outputs a tuple composed of the kidney segmentation mask $\textbf{K}$, the global scores $\textbf{p}$, and the region descriptors $\textbf{R}$

### Segmentation, Classification, and Detection CNN (SCD-CNN)

The proposed system is a hybrid architecture based on Mask R-CNN [20] for segmentation and Faster R-CNN [21] for region-based pathology detection. The choice of these networks allows us to share the most part of their architectures while efficiently solving kidney segmentation and region detection and is consistent with the results presented in [11] for a similar task (thyroid nodule detection). Furthermore, it is worth noting that we keep the boundary between the two architectures because we have information to segment the kidney, but we lack region masks to perform pixel-wise segmentation of the local pathologies. To be more specific, our system is built on a ResNet-50 backbone [43], which has been shown to be the most efficient backbone for image-based pathology classification in US [44]. In addition, we rely on Faster R-CNN and Mask R-CNN pre-trained with this backbone in Pytorch [45]. The backbone module is followed by two Feature Pyramid Networks, FPN, [46], one for kidney segmentation and the other for region-based local pathology detection. Figure 4 illustrates the detailed architecture of the network. Each component of the SCD-CNN is described below.

1. We use ResNet-50 backbone to compute a multi-scale representation of the input images, composed of 4 maps $\textbf{T}_l \in \mathbb {R}^{H_l\times W_l \times C_l}, l=1...4$, each one at a given spatial resolution $H_l=H/2^l; W_l=W/2^l$ defined by the accumulated spatial stride of the sub-network until its corresponding layer (e.g., stride=2, 4, 8, and 16), and with a given number of channels $C_l=128\cdot 2^l$ (e.g., 256, 512, 1024, and 2048). Our two FPNs receive these tensors as inputs and transform them into a set of multi-scale feature maps, each one specifically tailored for a task of interest: $\textbf{F}^{image}=\{\textbf{F}^{image}_l \in \mathbb {R}^{H_l\times W_l \times C}\}$ for kidney segmentation and $\textbf{F}^{region}=\{\textbf{F}^{region}_l \in \mathbb {R}^{H_l\times W_l \times C}\}$ for region-based local pathology detection. The multi-resolution approach (image-based and region-based features) will allow our system to exploit coarse-to-fine granularities in the image (e.g., using RoI-Pooling layers [47]) and only requires that the number of channels *C* is fixed along the scales (in our case, $C=C_0=256$ channels).
2. The kidney segmentation branch, inherited from Mask R-CNN, generates a binary mask $\textbf{K} \in \mathbb {R}^{H \times W}$ defining the region of the 2D image corresponding to the kidney, after performing the kidney detection with a Region Proposal Network over the FPN features $\textbf{F}^{image}$. This branch is trained using the multi-task loss $\mathcal {L}_{seg}$ proposed in the original Mask-RCNN paper [20].
3. The image-based classification branch analyzes the top ResNet-50 feature tensor, $\textbf{T}_4$, and outputs an image-based pathology probability vector $\textbf{p}^i = \{p^i_k\}, \ k \in [0,P]$ being *P* the total number of considered pathologies (both global and local). In particular, $k=0$ is reserved for the healthy category, the range $k \in [1,L]$ corresponds with the *L* local pathologies, and $k \in [L+1,P]$ with the *G* global ones. Hence, $P=G+L$ (see Table 1 and, in our particular case, $L=4$ and $G=2$).
  It should be noticed that the classification system does not only focus on the kidney area, because some of the pathologies are based on echoic differences between the kidney and its surroundings (they need more global contexts). Therefore, we are not using the segmentation branch to define the RoI for the classifier, but to regularize its operation. It is also worth mentioning that this classification branch is trained using a $\mathcal {L}_{clas}$ that in turn accumulates a set of $P+1$ binary cross-entropy losses, each one associated with one pathology (and the healthy category), to take into consideration that a single clinical case may present several concurrent pathologies.
4. The region-based detection branch produces a description of each clinical case through a set of *N* regions in which local pathologies have been detected. It is based on a Faster R-CNN object detection module and, for each region $n \in [1,N]$ identified as containing a local pathology, produces a 6-d region descriptor $\textbf{r}_n$, which has the following form:
  1 $$\begin{aligned} \textbf{r}_n=\left[ x^{min}_n \ x^{max}_n \ y^{min}_n \ y^{max}_n \ id_n \ s_n\right] \end{aligned}$$
  where the first 4 elements represent the coordinates of the bounding box containing the local pathology, $id_n \in [1,L]$ the identifier for the category of the local pathology, and $s_n$ the score for the local pathology contained in the region (in the form of a probability). Hence, each detected local region is associated with just one local pathology (the one with the maximum probability), and the region descriptor contains an indicator of this pathology and its predicted probability. This branch is trained through a multi-class loss $L_{reg}$, described in detail in [21]. The obtained region representations $\textbf{r}^n$ are finally stacked to form a matrix $\textbf{R} \in \mathbb {R}^{N \times 6}$. It is noteworthy that *N* varies from one clinical case to another, as only those candidate detections in which the value $s_n$ is above a threshold are considered.

Hence, for each clinical case $\textbf{C}$, our SCD-CNN outputs a triplet $\left\{ \textbf{K}, \mathbf {p^i}, \textbf{R}_{N\times 6}\right\}$, containing the kidney segmentation mask ($\textbf{K}$), the pathology probabilities predicted by the image-based classification branch ($\mathbf {p^i}$), and the description of the detected regions potentially exhibiting local pathologies ($\textbf{R}_{N\times 6}$), respectively.

### Fusing Image- and Region-Based Predictions: Diagnosis Generation Module

**Fig. 5:**
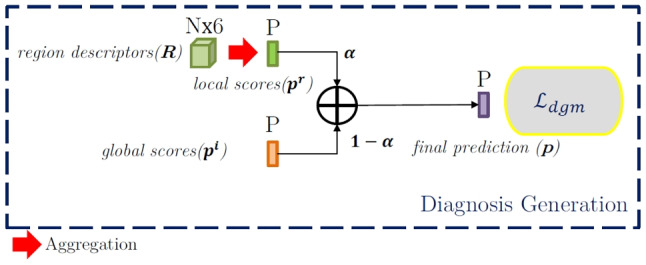
Block diagram of the Diagnosis Generation Module. The region-based pathology scores $\textbf{R}$ are aggregated per pathology (resulting the vector $\textbf{p}^r = \{p^r_k\}, \ k \in [0,P]$) and combined with the image-based ones (denoted by $\mathbf {p^i}$) to compose the final tentative diagnosis of the clinical case

The Diagnosis Generation Module, depicted in Fig. 5, is responsible for combining image- and region-based information to make a tentative diagnosis of the kidney. To that end, our objective is to leverage the different granularities of the information in the 2D US renal images by fusing the information at two levels: (1) at an image level, considering the entire kidney and their surroundings (useful for the detection of both global pathologies and local ones, particularly those that involve a significant percentage of the kidney’s area), and (2) at a region level, considering local information at regions detected as potentially exhibiting local pathologies (this helps with detecting pathologies of smaller size, which may be difficult to notice at the image level). In doing so, we can effectively address the two-level taxonomy of our scenario.

The fusion process follows three stages, which are described in detail below: we first generate a probability vector associated with each considered local pathology relying on the region-based branch of the network; then we assign a healthy probability to the clinical case relying also on information from this branch; and finally, we combine the probabilities coming from the image- and region-based branches of the network.

First, it is necessary to define an aggregation mechanism to transform the scores of the local pathologies contained in $\textbf{R}_{N\times 6}$ into global image-level probability vector $\mathbf {p^r}$ of length *L* (the total number of local pathologies). We have considered several aggregation mechanisms:

- Max-aggregation: for each pathology consider the maximum probability among those provided by the detected regions (for the regions belonging to each category *k*).
  2 $$\begin{aligned} \mathbf {p^r}_k=\max _{n | id_n=k}\left( s_n\right) , \ \ k \in [1,L]. \end{aligned}$$
- Mean-aggregation: considering the mean of the probabilities of the detected regions for each pathology.
  3 $$\begin{aligned} \mathbf {p^r}_k=\frac{1}{N}\sum _{n | id_n=k}{\left( s_n\right) }, \ \ k \in [1,L]. \end{aligned}$$
- LME-aggregation (Log-Mean-Exp): it is a intermediate version between max- and mean-aggregation.
  4 $$\begin{aligned} \mathbf {p^r}_k=\log \left( \frac{1}{N}\sum _{n | id_n=k}{e^{s_n}}\right) , \ \ k \in [1,L]. \end{aligned}$$
- Area-aggregation: taking into account both the area and the probability of each detected region with the kidney area as a reference.
  5 $$\begin{aligned} \mathbf {p^r}_k=\frac{1}{\sum _{xy}{\textbf{K}}}\sum _{n | id_n=k} s_n h_n w_n, \ \ k \in [1,L], \end{aligned}$$
  with $h_n=y^{max}_n-y^{min}_n$ and $w_n=x^{max}_n-x^{min}_n$ and $\sum _{xy}{\textbf{K}}$ the kidney area, as the number of non-zero pixels in the binary mask.

We will comprehensively assess the performance of each aggregation method in “Assessment of the Aggregation Method”.

Second, in addition to the scores for each local pathology, we form the final vector $\mathbf {p^r}$ by adding:

- A score for a healthy kidney (first position in the vector). If a clinical case has a low score for every local pathology, its probability to be healthy must be high, and vice versa. Thus, the local probability for a clinical case to be healthy, $p^r_0$, is computed as
  6 $$\begin{aligned} p^r_0=1-\frac{1}{L} \sum _{k=1}^L {p^r_k} \end{aligned}$$
- The probabilities for global pathologies at the end of the vector, which are all set to zero: $p_k^r=0, \ k \in [L+1,P]$ as they are not considered in the local branch of our system.

This yields a $\mathrm {P+1}$-dimensional vector $\textbf{p}^r$ with the local probabilities of the different pathologies.

Once we have the two vectors of predictions, $\mathbf {p^i}$ and $\mathbf {p^r}$, coming from the image- and region-based branches of our system, we perform a convex combination to generate the fused vector with final predictions $\textbf{p} = \{ p_k \}, k \in [0,P]$:

7 $$\begin{aligned} \textbf{p}=\varvec{\alpha }\odot \mathbf {p^i}+\left( \textbf{1}-\varvec{\alpha }\right) \odot \mathbf {p^r}, \end{aligned}$$

where $0\le \varvec{\alpha }\le 1$ is a learnable parameter that controls the influence of global and local predictions in the fusion, setting their influences over the final system decision. The optimum values for $\varvec{\alpha }$ are learned through the loss $\mathcal {L}_{dgm}$ (in the form of a set of $P+1$ binary cross-entropy losses as $\mathcal {L}_{clas}$). We assume that $\varvec{\alpha }$ will take different values depending on the pathology, leading to a system adaptation to each particular disease.

In particular, we have considered two strategies to learn the values of the $\varvec{\alpha }$:

- **Category-level fusion**: the first strategy considers a global set of category-dependent $\alpha _k$, which remain fixed for every image in the database. This approach provides an interpretable result of the importance of the global and local predictions for each category of the taxonomy, i.e., a local category *k* defined by small regions will have a corresponding smaller value of the $\alpha _k$ parameter than the same local category characterized by bigger regions. The fusion parameter $\varvec{\alpha }$ is defined as a parameter of the neural network and is learned through the loss $\mathcal {L}_{dgm}$.
- **Attention-based fusion**: attention mechanisms allow networks to focus on specific information in each situation. In our case, we propose to use attention to automatically set the value of $\varvec{\alpha }$ according to the particular features of each clinical case. This strategy allows practitioners to analyze each case considering the specific $\varvec{\alpha }$ weights estimated by the CAD system. In addition, we can still perform a category-level examination by analyzing the distributions of the $\varvec{\alpha }$ parameter over the entire dataset. In particular, we have proposed a simple attention module in which $\varvec{\alpha }$ is predicted by a linear layer working over the concatenation of global and local predictions:
  8 $$\begin{aligned} \varvec{\alpha } \propto W_{att} [p^i; p^r] + b_{att} \end{aligned}$$
  where the parameters $W_{att}$ and $b_{att}$ are learned using the loss $\mathcal {L}_{dgm}$.

Both fusion strategies and the obtained values of $\varvec{\alpha }$ will be deeply discussed in “Ablation Study and Analysis of the Fusion Parameters”.

Finally, the losses used to train our SDN-CNN deserve a comment. The system is trained through a multi-class loss $\mathcal {L}$ that incorporates a significant number of losses, as shown in Figs. 4 and 5 and described above. We expect multi-task acting as a regularizer that allows learning a better global CAD system, in the sense that each one of the branches benefits from the knowledge learned by the rest, specially the image-based classification and region-based detection branches. For simplicity and to avoid biases towards any specific task, we have used a simple sum of the corresponding losses so $\mathcal {L}=\mathcal {L}_{seg}+\mathcal {L}_{clas}+\mathcal {L}_{reg}+\mathcal {L}_{dgm}$. With this approach, the whole system is trained in an end-to-end basis, with all the losses contributing in the same degree to the total loss.

## Results

### Experimental Setup

**Fig. 6:**
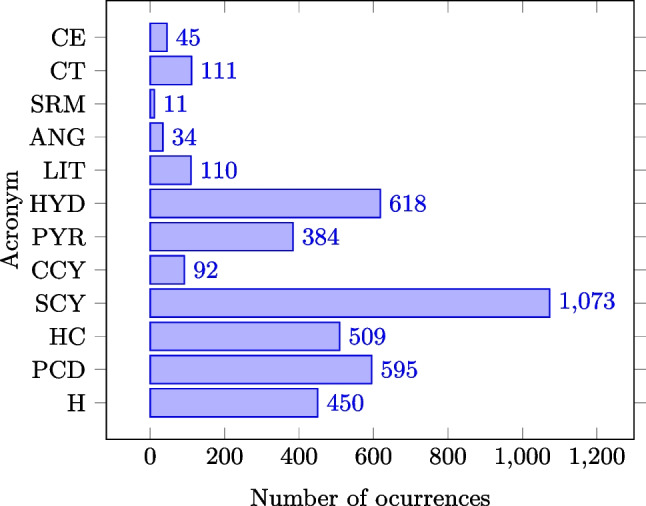
Distribution of the pathologies in the dataset. SCY and CCY compose the “cyst” category (C), and LIT, ANG, SRM, CT, and CE are grouped in the “others” category (O)

To assess URI-CADs, we have built a 2D US imaging database containing the kidney location and complete diagnosis of 1985 images (with 450 healthy and 1535 pathological kidneys), annotated by two experienced nephrologists from Hospital Ramón y Cajal, Madrid (Spain), through the annotation procedure described in Subsection 3.2. The dataset is publicly available with the goal of promoting future developments and research in the field^1^. Proportions between healthy and pathological cases are those common in patients that are referred by physicians in primary care to nephrologists. The annotations for each clinical cases include: a polygonal segmentation mask of the kidney, indicators of presence of global pathologies, and also indicators and bounding boxes of the local lesions (when present). The distribution of the pathologies in the database is shown in Fig. 6. Due to the scarcity of samples of several of the pathologies, and for practical purposes, we have decided to group some of them according to these guidelines: 1) some pathologies are grouped if they have something in common (for example, the category cyst does not distinguish between simple and complicated cysts); 2) some categories are grouped if their individual number of samples is insufficient to properly train the system: the category others (O) groups all the pathologies whose number of samples is very low: lithiasis, angiomyolipoma, solid renal mass, cortex thinning and cortex schar. This leads to a final set of P=6 categories, two global: hyper-echoic cortex (HC) and poor corticomedullary differentiation (PCD), and four local: cyst (C), stone (S) , hydronephrosis (HYD), and others (O). We have demonstrated that the uneven distribution of the dataset has no negative impact in the results and that our system is not biased to any kind of errors through the error analysis (see “Error Analysis and Discussion”).

We have followed a 5-fold cross-validation strategy in our experiments, using a three folds for training, one for validation, and one for testing in each repetition. Realistic data augmentation techniques, supervised by the nephrologists (rotation, gamma adjustment, translation, and zoom), were randomly applied to the images during training in all the experiments. Our code was developed using Python (we employed Pytorch [45], torchvision, and OpenCV for data augmentation).

We have selected two broadly adopted performance metrics to evaluate our system capability to segment the kidney area: IoU and Dice coefficients, and other specific two to assess its capability to diagnose: (1) the area under the Sensitivity-Specificity (SP) Receiver-Operating Characteristic (ROC) curve: $\textrm{AUC}_{\mathrm {SENS-SP}}$, for each category, by considering a binary problem “category vs non-category,” indicating how well the images are ranked in terms of the soft score for a specific pathology provided by the system under evaluation, and specially useful for medical applications [48, 49]; and (2) the specificity at a sensibility of 95% ($\textrm{SP}_{\textrm{SENS}-95}$) [48, 49]. This last metric is particularly helpful if the proposed system is used as a filter for referring patients from primary care to specialists, as it indicates how many of the non-referred cases are truly healthy, or equivalently (if we take its complementary value), how many of the healthy (non relevant) cases will be referred, assuming a minimum referral rate of 95% for pathological (relevant) cases. In this way, we can assess the capacity of our system for each marginal classification problem and extract conclusions disregarding the potential database imbalance.

The experiments in this section are organized as follows: first, we select the optimum aggregation method for our system in “Assessment of the Aggregation Method”. Then, we analyze and discuss the results obtained by the proposed fusion strategies in “Ablation Study and Analysis of the Fusion Parameters”. Finally, we present the results of the proposed system in terms of segmentation and classification performance, in comparison with those of several state-of-the-art systems, in “Comparison with the State-of-the-Art”.

### Assessment of the Aggregation Method

This section focuses first on determining the best-performing aggregation method for the Diagnosis Generation Module among the methods proposed in “Fusing Image- and Region-Based Predictions: Diagnosis Generation Module”. The validation process is performed using the first fold of our 5-fold cross-validation strategy, assuming that the optimal hyperparameters for this fold will be also suitable for the remaining data, and minimizing the risk of overfitting. In fact, our method turns out to be quite robust, and results in distinct folds are not significantly different.

**Table 2:** Average $\textrm{AUC}_{\mathrm {SENS-SP}}$ for the different aggregation methods over the considered local categories. Results are computed using in the first fold as test set

**Type of aggregation**	$\textbf{AUC}_{\mathbf {SENS-SP}}$ **(%)**
**Max-aggregation**	$\mathbf {80.97}$
**Mean-aggregation**	79.31
**LME-aggregation**	79.28
**Area-aggregation**	80.59

Regarding the aggregation method, Table 2 shows the $\textrm{AUC}_{\mathrm {SENS-SP}}$ for the local categories of the database. According to the results, max-aggregation is the best performing aggregation method (with similar performance to the area-aggregation method). It turns out to be slightly better to rely on the detected regions with high scores to decide on each local pathology. Hence, max-aggregation will be used from now on the rest of the experiments.

**Table 3:** Ablation study: different fusion strategies for the system proposed in this paper. Ultrasound kidney classification results in terms of $\textrm{AUC}_{\mathrm {SENS-SP}}$ (%) for each category in the taxonomy. Categories are labeled as global (G) or local (L)

	**Multi-pathological**							**Binary (healthy/pathological)**
**Method**	**HC** (G)	**PCD** (G)	**C** (L)	**PYR** (L)	**HYD** (L)	**O** (L)	**Average**	**H**
**URI-CADS-I**	77.33	82.57	73.30	71.10	89.75	66.90	76.83	85.34
**URI-CADS-R**	−	−	72.72	79.27	87.08	51.01	72.52	77.53
**URI-CADS-C**	76.56	81.70	78.43	82.00	91.69	67.34	79.62	87.21
**URI-CADS-Att**	$\mathbf {78.65}$	$\mathbf {84.15}$	$\mathbf {79.59}$	$\mathbf {86.61}$	$\mathbf {93.04}$	$\mathbf {69.32}$	$\mathbf {81.90}$	$\mathbf {87.41}$

### Ablation Study and Analysis of the Fusion Parameters

This section is devoted to perform an ablation study and analyze the optimum values of the fusion parameter for each one of the fusion strategies presented in “Fusing Image- and Region-Based Predictions: Diagnosis Generation Module”, for the sake of explainability.

Table 3 shows an ablation study of several ablated versions of our system, namely URI-CADS-I, a version of our system that only includes the image-based branch; URI-CADS-R, a version that only incorporates the region-based branch; and the two fusion strategies: URI-CADS-C, using the category-based fusion strategy and URI-CADS-Att, employing the attention-based fusion.

**Table 4:** Values of the fusion parameters per category. *URI-CADS-C: averaged in the five different folds, a set of values per fold. *URI-CADS-Att: averaged for each clinical case, one set of values per each clinical case

**Method**	$\mathrm {\varvec{\alpha }_{{\textbf {H}}}}$	$\mathrm {\varvec{\alpha }_{{\textbf {HC}}} (G)}$	$\mathrm {\varvec{\alpha }_{{\textbf {PCD}}} (G)}$	$\mathrm {\varvec{\alpha }_{{\textbf {C}}} (L)}$	$\mathrm {\varvec{\alpha }_{{\textbf {PYR}}} (L)}$	$\mathrm {\varvec{\alpha }_{{\textbf {HYD}}} (L)}$	$\mathrm {\varvec{\alpha }_{{\textbf {O}}} (L)}$
**URI-CADS-C**	1.0000	1.0000	1.0000	0.9904	0.7539	1.0000	0.8103
URI-CADS-Att*	0.9833	0.8637	0.8281	0.9166	0.6563	0.8329	0.6736

Results in Table 3 account for the need of multiple resolutions in our ultrasound renal imaging diagnosis task. Our URI-CADS-C and URI-CADS-Att approaches successfully integrate the two-level information to provide notably better diagnosis for almost all the categories in except of the “others.” For this last category, it is very difficult to set a proper value of $\mathbf {\alpha }$, as due to the varying nature, appearance, and shape of the different pathologies aggregated into this category. In addition, it is remarkable that even in the case of global pathologies (HC and PCD) that, *a priori*, are detected through the global classifier, the URI-CADS-Att multi-task approach performs better than the URI-CADS-I ablated version which takes into account only the global information of the clinical case. Although this result may seem surprising, the rationale behind is that our attention module is being able to modulate the scores of global pathologies by analyzing the information of the remaining ones (see Eq. (8)).

Furthermore, despite the URI-CADS-R (the region-based ablated version) results show a more modest performance (it is the most challenging task), their integration with the global predictions of the network substantially improves the performance (around 3% of AUC for every category of the taxonomy except the global pathologies and more than 6% in the case of cysts or pyramids).

The fusion parameters per category for each one of the fusion strategies are gathered in Table 4. Their values provide meaningful insights regarding the significance of each type of information for the diagnosis. In the case of the healthy vs. pathological diagnosis, global (image-level) information clearly dominates over local information. According to the nephrologists, a kidney is considered healthy when the cortex and sinus can be properly distinguished, its shape is elliptical, and it does not present any lesion. These features can be inferred from the complete ultrasound image (i.e., from the global view), so its fusion parameters tend to one. The same reasoning can be employed for the global features (HC and PCD). However, in the case of the local pathologies, the fusion parameter value depends on the area occupied by the pathology: on the one hand, cysts (C) and hydronephrosis (HYD) present greater areas, so they can be inferred from the local view and their fusion parameters tend to one, on the other hand, pyramids (PYR) and other pathologies (O) have smaller sizes in general, thus, balancing the two perspectives of the proposed approach results in the best performance. Even in such cases, the weight of the global view becomes dominant, because, thanks to the regularization ability of the multi-task approach, the backbone can learn some activations that indicate the presence or absence of local lesions.

Furthermore, regarding the fusion parameters resulting in URI-CADS-C and URI-CADS-Att approaches, the values for the latter are less extreme. Depending on the clinical case, the system can balance the diagnosis towards the region-based decision (for example, for small local pathologies). In addition, it is remarkable that the URI-CADS-Att approach applies a non-zero weight to the local view of the global pathologies ($\alpha _{HC}$ and $\alpha _{PCD}$ parameters are not zero). As we have already mentioned, our attention-based module is able to re-modulate the score of the global pathologies through the analysis of the remaining ones (e.g. by reducing the scores of global categories if some local pathologies are found in the lesion). This very interesting behavior cannot be achieved by the category-level fusion, which fixes the $\varvec{\alpha }$ values for the entire dataset.

The previous point, together with the ability of adapting the fusion to the particular characteristics of each clinical case, allows URI-CADS-Att to yield an average performance improvement of a $2.28\%$ over URI-CADS-C and become the reference model to be used in the rest of this paper.

## Discussion

In order to provide more insight into the capabilities and limitations of the proposed system, we first compare its results with the ones from the state of the art in “Comparison with the State-of-the-Art” and then examine the errors made by the system by analyzing the results of each individual module (segmentation and classification) in “Error Analysis and Discussion”.

### Comparison with the State-of-the-Art

The goal of this section is to assess the proposed system in comparison to relevant systems in the literature. It is noteworthy that, in comparison with the systems presented in “Introduction”, which reported results using test sets of tens or few hundreds of images, our performance metrics are obtained over a dataset of 1985 US images using a 5-fold cross-validation strategy.

The experiments are organized into two blocks: segmentation and classification.

**Table 5:** Comparison of US kidney segmentation results with the state-of-the-art methods in terms of IoU and Dice coefficients in several datasets, which are described by their size

**Database (# of images)**	**DB** [32] ($\textbf{80}$)	**DB** [28] ($\textbf{50}$)	**DB** [31] ($\textbf{289}$)	**DB ours (** $\textbf{1985}$**)**
**Method**	**IoU/Dice** **(%)**			
**Deeplabv3+** [50]	$-/92.8$	$88.69/-$	81.87/89.85	81.80/89.34
**CT2US** [32]	$-/95.2 \ (+2.4)$	−	−	−
**SDFNet** [28]	−	$91.24 \ (+2.55)/-$	−	−
**Bnet** [31]	−	−	$87.29 \ (+5.42)/93.03 \ (+3.18)$	−
**TN-SCUI2020** [51]	−	−	−	$79.31 \ (-1.49)/87.23 \ (-2.11)$
**URI-CADS-Segmentation**	−	−	−	$\mathbf {84.99 \ (+3.19)}/\mathbf {91.23 \ (+1.89)}$
**URI-CADS**	−	−	−	$81.41 \ (-0.39)/89.38 \ (+0.04)$

#### 2D Kidney Ultrasound Segmentation

Although segmentation is not the focus of our approach, in this first set of experiments, we aim to assess the performance of our method to segment the kidney in US images. We have compared our approach to the most relevant state-of-the-art systems, showing their results in the reported scenarios. To make this comparison meaningful, we also provide the performance of Deeplabv3+ [50], as it has been reported in all cases and constitutes the reference that allows comparing results over different datasets. Additionally, we have also included the performance in our scenario of the winner solution of the thyroid nodule segmentation and Classification grand challenge TN-SCUI2020, which performs segmentation and classification in US images using the same architecture [51]. All the results are presented in terms of IoU and Dice coefficient in Table 5. It should be noted that, to perform an ablation study, we also include a simplified version of our system (URI-CADS-Segmentation) which only detects and segments the kidney and not provides a diagnosis to evaluate if improvements in classification and detection come at the cost of a slight decrease in segmentation performance.

As observed in Table 5, the version of our system that focuses on kidney detection and segmentation (URI-CADS-Segmentation) provides a 3% improvement in terms of IoU compared to Deeplabv3+ in our dataset. This allows us to conclude that the kidney detection approach (with subsequent segmentation through Mask R-CNN) is more effective for our segmentation task. Furthermore, the performance of Deeplabv3+ in our scenario is always lower than that on other datasets (sometimes by a significant margin [28]), which reveals that our database is not only larger than the others, but also more challenging. In general, the performance of our proposed system is similar to that achieved by the rest of the compared methods in the state-of-the-art, even when the focus of our approach is not segmentation, which remains an auxiliary task. Indeed, when including the global and local branches for classification (URI-CADS-C and URI-CADS-Att), the performance decreases slightly (about 3%) in comparison with the only-segmentation system (URI-CADS-Segmentation). However, this slight decrease in the auxiliary task (segmentation) is compensated by a significant improvement in the main objective of our system: pathology classification (as will be seen in the next section).

**Fig. 7:**
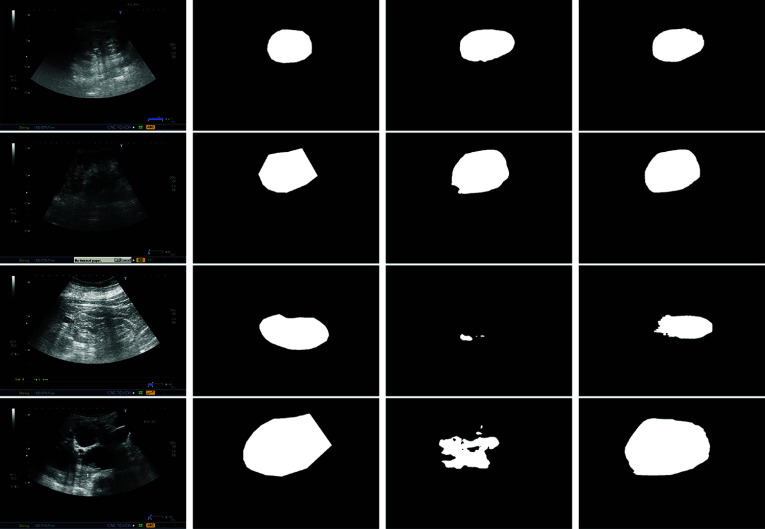
Illustrative examples of kidney US segmentation. First column: original volume; second column: ground-truth segmentation; third column: Deeplabv3+ and fourth column: URI-CADS

Finally, we present some illustrative examples of kidney segmentation with our system and Deeplabv3+ in Fig. 7. The results reveal that Deeplabv3+ struggles to segment PCD kidneys (due to their low contrast with the background) and poly-cystic ones (because their unique appearance compared to the rest of the kidneys). On the other hand, URI-CADS and URI-CADS-Segmentation (not included in the figure), produce more consistent and accurate results by incorporating the detection process. It is worth noting that our system successfully detects all but one kidney in the database, indicating that the main decrease in performance is primarily attributed to the segmentation of the kidney boundaries, particularly in challenging ones.

#### 2D Ultrasound Kidney Classification

In order to evaluate the performance of our proposed 2D US kidney classification system, we have compared it to the state-of-the-art by Sudharson and Kokil [16], which deals with a similar scenario to ours (multi-class classification: normal, cyst, stone, and tumor, in their case), with promising results in their database. It is worth noting that in their scenario, each clinical case belongs to only one category of the taxonomy, whereas in our scenario, the same clinical case can present one or more pathologies (images are sampled from real-world studies without bias).

In the work of Sudharson and Kokil [16], they use an ensemble of 3 CNNs pre-trained on ImageNet to classify images into a single class out of multiple classes (single-label multi-class classification). They train one SVM per CNN, where each SVM is trained on a set of features extracted from the corresponding CNN. However, in our scenario, where a single image can exhibit multiple pathologies, we have adapted their approach by training one binary SVM per pathology category. In addition, Sudharson and Kokil combine the hard scores of the SVMs using majority voting, whereas in our implementation of their system, we found that averaging the soft scores yielded better results. We refer to this adapted approach as SUDHARSON-ORIG and also propose an improved version (SUDHARSON-IMP) where each CNN is fine-tuned in our task before averaging their scores. In this way, we can demonstrate that our approach outperforms an ensemble of several CNNs with a single multi-task architecture which concurrently addresses the tasks of segmentation, classification and pathology detection in US images.

Furthermore, we have also included in our comparison the results of the winner method of the TN-SCUI2020 Grand Challenge [51], which was initially designed for thyroid nodule segmentation and binary classification in US images. In order to adapt this method to our scenario, we have replaced the binary classification loss with our set of binary cross-entropy losses to address our multi-class classification task.

The results for 2D kidney US classification are presented in Table 6. We have also conducted an ablation study, in which we analyzed the performance of our method when relying only on image-level features (URI-CADS-I) or region-level features (URI-CADS-R), just before the Diagnosis Generation Module, which is the one that combines both scores to provide the final tentative diagnosis. This study aimed to understand how the region-based branch improves the overall classification performance of the system.

**Table 6:** Comparison of US kidney classification results with state-of-the-art methods in terms of AUC-PR and SP-95 for each category in the taxonomy

		**Multi-pathological**							**Binary (healthy/pathological)**
**Method**	**Measurement**	**HC**	**PCD**	**C**	**PYR**	**HYD**	**O**	**Average**	**H**
**SUDHARSON-ORIG** [16]	$\textrm{AUC}_{\mathrm {SENS-SP}}$ (%)	47.70	50.80	49.76	48.39	50.02	49.37	49.34	47.14
	$\textrm{SP}_{\textrm{SENS}-95}$ (%)	6.04	8.20	5.49	4.39	4.55	3.70	5.40	6.71
**SUDHARSON-IMP**	$\textrm{AUC}_{\mathrm {SENS-SP}}$ (%)	59.29	63.95	52.10	53.99	67.79	58.18	59.22	64.37
	$\textrm{SP}_{\textrm{SENS}-95}$ (%)	10.43	12.67	6.90	11.57	9.17	11.56	10.38	29.19
**TN-SCUI2020** [51]	$\textrm{AUC}_{\mathrm {SENS-SP}}$ (%)	70.30	75.34	73.89	74.91	85.85	64.54	74.14	77.39
	$\textrm{SP}_{\textrm{SENS}-95}$ (%)	20.86	29.68	15.88	12.50	38.13	11.97	21.50	27.11
**URI-CADS**	$\textrm{AUC}_{\mathrm {SENS-SP}}$ (%)	$\mathbf {78.65}$	$\mathbf {84.15}$	$\mathbf {79.59}$	$\mathbf {86.61}$	$\mathbf {93.04}$	$\mathbf {69.32}$	$\mathbf {81.90}$	$\mathbf {87.41}$
	$\textrm{SP}_{\textrm{SENS}-95}$ (%)	$\mathbf {29.28}$	$\mathbf {43.63}$	$\mathbf {28.19}$	$\mathbf {48.19}$	$\mathbf {63.19}$	$\mathbf {14.05}$	$\mathbf {37.76}$	$\mathbf {60.59}$

The proposed system demonstrates superior performance compared to Sudharson’s 2D US classification systems, as evidenced by its notable margin of improvement in AUC (+20%) in every category of our database. When compared to the TN-SCUI2020 system, which is designed for nodule detection, our approach shows a relative improvement of 10% in terms of AUC in binary classification and approximately 6% in multi-pathological classification. These results suggest that a multi-task regularized framework, such as the one proposed in this paper, can effectively leverage both local and global information extracted from images in a challenging scenario. Furthermore, our system’s use of both kidney location and segmentation information through the detection and segmentation branch was found to be crucial in differentiating pyramid pathologies from other hypo-echoic areas, such as cysts.

In terms of real-world applicability, the proposed system shows promising results. The binary pathological vs. healthy classification achieved an AUC of 87% and a $\textrm{SP}_{\textrm{SENS}-95}$ value of 60%. This suggests that, when used in a primary healthcare setting for identifying cases that require referral to specialists, less than 40% of healthy kidneys would be referred while ensuring that 95% of pathological cases are correctly referred. Additionally, the multi-class classification results for most categories are around 80% AUC, which is considered to be helpful for expert practitioners.

### Error Analysis and Discussion

**Fig. 8:**
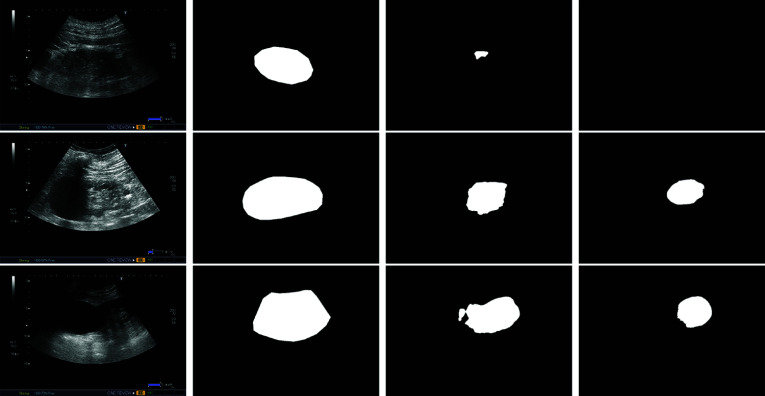
Illustrative examples of the most serious errors made by the 2D kidney US segmentation system. First column: original image; second column: ground-truth segmentation; third column: Deeplabv3+ and fourth column: URI-CADS

Figure 8 shows some illustrative examples of the most serious errors of the kidney segmentation branch. These errors mainly occur when there is a lack of contrast between the kidney and the background, such as in cases of hyper-echogenia or poor corticomedullary differentiation. Additionally, cases with ambiguities, such as other organs that resemble the kidney, can also cause errors (although these cases are not common in our database). As we already mentioned, the system only failed to detect the kidney in one case. Moreover, as can be inferred from the other examples, even in these worst cases, the system provides a more regularized solution than Deeplabv3+. These extreme cases have associated an error in the healthy-pathological diagnosis, but, when the segmentation covers a significant kidney area (above 0.3 of IoU), the diagnosis is accurate.

**Fig. 9:**
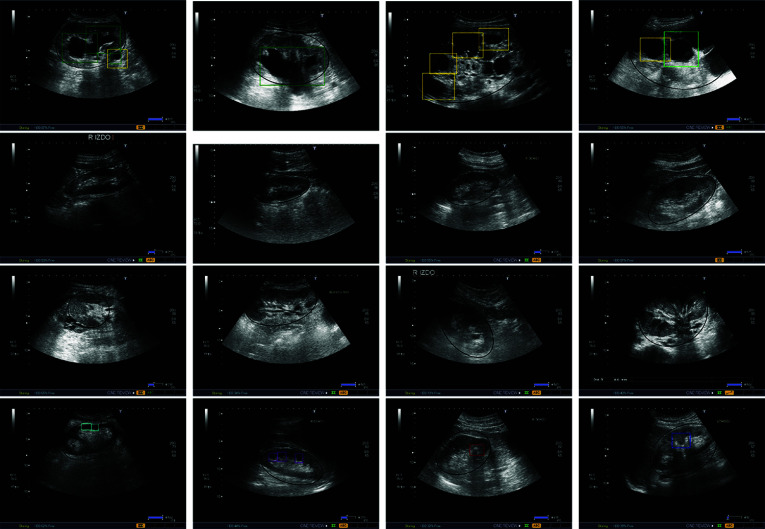
Illustrative examples of the consistency of the binary healthy vs. pathological classification. First row: true positives (pathological kidneys correctly classified); second row: true negatives (healthy kidneys correctly classified); third row: false positives (healthy kidneys incorrectly classified as pathological), and fourth row: false negatives (pathological kidneys incorrectly classified as healthy). Color boxes mark off the local pathologies: cysts and hydronephrosis in the first row, and angiomyolipoma, pyramid, lithiasis, and solid renal mass in the last row

Regarding the binary classification of the US renal images (the main objective of our system), it is important to ensure that the system’s output is consistent. This means that the scores given to clearly pathological cases are high, the scores given to clearly healthy cases are low, false positives (healthy kidneys that are incorrectly classified as pathological) do not have a clear differentiation between the renal cortex and sinus, and false negatives (pathological kidneys that are not detected) have unclear or difficult pathologies. Figure 9 illustrates some representative examples of these categories. For our system, healthy kidneys with a clear differentiation between the renal cortex and sinus and no visible pathologies are classified as healthy, while kidneys with obvious pathologies such as hydronephrosis and cysts are classified as pathological. Additionally, the errors in our system come from the classification of some kidneys with subtle global pathologies (HC and PCD) as false positives and some kidneys with small, barely visible local pathologies as false negatives (both are difficult cases). Overall, our system demonstrates robust and consistent performance.

## Conclusion

In this paper, we present URI-CADS, a fully automated computer-aided diagnosis system for ultrasound renal imaging that concurrently performs kidney segmentation and tentative diagnosis of 2D US renal images within a single framework.

The system aims to achieve two objectives: z91) to assist non-expert practitioners in primary healthcare by improving clinical workflow and reducing specialist workload; and 2) due to the comprehensive taxonomy used, it could become a helpful tool for training and supporting expert practitioners, reducing human biases, and providing meaningful insights.

Our experimental results demonstrate that a joint approach to segmentation, global classification, and local detection of pathologies in each clinical case notably improves the diagnosis performance compared to state-of-the-art methods. Our strategy outperforms more complex systems based on ensembles of CNNs and the state-of-the-art hybrid system for segmentation and classification of thyroid nodules. Our proposed multi-task regularization is crucial for performance improvement as each branch learns from the other branches, particularly the classification branch. Additionally, the system provides kidney segmentation results comparable to the state of the art. To the best of our knowledge, this is the first time that such a comprehensive automatic analysis of ultrasound renal clinical cases has been performed with notable performance.

Moreover, we found that our system’s results are consistent, which is crucial for the future deployment of CAD systems in the healthcare system. Our method can be applied to primary health as a first filter to reduce specialist workload and accelerate diagnosis, or used by expert practitioners to support their hypotheses and receive meaningful suggestions during their daily activity. In addition, we have established a benchmark for ultrasound renal imaging analysis (segmentation, binary diagnosis and multi-pathological diagnosis) by publicly releasing our dataset, thus helping to promote the future research in the field.

Our envisaged further research includes enriching the current database, especially with pathologies in the “others” category, and extending the system to other scenarios. With the completion of the database, the 69% AUC that we obtained for the “others” category could be substantially improved, and even a marginal detection of pathologies under the “others” category could be proposed. Additionally, our proposed methodology could be extended to other scenarios to prove its versatility. Finally, the diagnosis of renal clinical cases often includes a kidney shape/size study. We will research how to incorporate a shape/size description into our system to enhance the results while maintaining its end-to-end trainability.

## Data Availability

The anonymised data and the code that support the findings of this study are available in the following repository: https://github.com/miguel55/URI-CADS/.

## Footnotes

1 Code and link to the dataset repository are available at https://github.com/miguel55/URI-CADS/
